# Clinical research of drug-coated balloon after rotational atherectomy for severe coronary artery calcification

**DOI:** 10.1186/s12872-023-03071-8

**Published:** 2023-01-21

**Authors:** Haozhe Dong, Yingguang Shan, Shenzhen Gong, Ran Li, Yiming Li, Xupeng Lu, Guoju Sun

**Affiliations:** Department of Cardiovascular Medicine, The First Affiliated Hospital of Zhengzhou University, Zhengzhou, 450052 Henan China

**Keywords:** Drug-coated balloon, Coronary calcified calcification, Rotational atherectomy, Drug-eluting stent

## Abstract

**Background:**

Current research results show that drug-coated balloons (DCB) have unique advantages in the treatment of in-stent restenosis, small vessel disease, bifurcation lesions, and de novo lesions, but the data regarding rotational atherectomy (RA) followed by DCB treatment in calcified lesions, especially severe coronary artery calcification (CAC), are limited.

**Methods:**

A retrospective study was conducted on 318 individuals with severe CAC who underwent RA-assisted PCI at the First Affiliated Hospital of Zhengzhou University from May 2018 to July 2021. Among them, 57 patients (RA/DCB group) were treated with DCB, and 261 patients (RA/DES group) were treated with drug-eluting stents (DES). The two groups' clinical baseline data, lesion characteristics, intraoperative complications, in-hospital adverse events, and major adverse cardiovascular and cerebrovascular events (MACCE) were compared throughout the follow-up period.

**Results:**

The baseline clinical data, intraoperative complications, and in-hospital adverse events were not significantly different between the two groups. The anatomical categories in the RA/DES group were more complex and included left main coronary disease, bifurcation disease, and multivessel disease. Although target lesion revascularization (13.79% vs. 7.02%) and MACCE (18.77% vs. 12.28%) occurred more frequently in the RA/DES group than in the RA/DCB group, there was no statistically significant difference (*p* > 0.05). Multivariate Cox regression analysis showed that bifurcation lesions (HR 2.284, 95% CI 1.063–4.908, *p* = 0.034), total length of DCB/DES (HR 1.023, 95% CI 1.005–1.047, *p* = 0.014) and SYNTAX score (HR 1.047, 95% CI 1.013–1.082, *p* = 0.006) were independent risk factors for MACCE during the follow-up period.

**Conclusion:**

Drug-coated balloon treatment after rotational atherectomy appears safe and effective in selected severe coronary artery calcification.

## Introduction

Even in the era of new-generation drug-eluting stents (DES), severe coronary artery calcification (CAC) remains a significant challenge for percutaneous coronary intervention (PCI) [1]. CAC is difficult to adequately predilate due to resistant plaque burden and surface irregularities, which may result in failed device delivery or incomplete stent expansion. Moreover, calcified plaques could increase technical difficulties and postoperative complications such as restenosis and thrombosis [2]. Therefore, adequate lesion preparation is a prerequisite for severely calcified lesions. Rotational atherectomy (RA) is the most widely used approach to modify the physical attributes of calcified plaques in preparation for angioplasty and stent deployment and subsequently optimize postprocedural results [3]. RA followed by DES implantation is currently the standard PCI strategy for severe CAC. Drug-coated balloons (DCB) provide an alternative treatment strategy for coronary artery disease by homogeneously releasing antiproliferative drugs to the lesion site during single balloon inflation and carry several anticipated benefits over DESs, including the absence of metallic stents and shorter duration of dual antiplatelet therapy (DAPT) [4]. Furthermore, DCBs show potential applications in some anatomical scenarios. These include in-stent restenosis, bifurcations, and small vessels, where stenting can lead to poor outcomes [5]. For severely calcified lesions, using RA prior to DCB therapy is thought to reduce the calcification burden and thus improve drug penetration into the vessel wall. Due to the lack of clinical studies, it is unclear whether RA followed by DCB implantation can be used as an alternative treatment strategy for severely calcified lesions. This study aimed to compare the efficacy and safety of RA/DCB and RA/DES in treating severe CAC and provide new evidence for addressing this stent-less strategy.

## Methods

### Study population

From May 2018 to July 2021, consecutive patients who underwent RA followed by DCB or DES at the First Affiliated Hospital of Zhengzhou University (Henan, China) were enrolled. Only patients with severe CAC were eligible for the study, defined as radiopacities noted without cardiac motion before contrast injection and generally involving both sides of the arterial wall. The exclusion criteria were as follows: (1). Myocardial infarction within four weeks; (2). Unstable hemodynamics or cardiogenic shock; (3). Limited long-term prognosis due to other conditions; (4). Coronary artery bypass graft stenoses; (5). Lesion angulation > 90°; (6) Lesions used DES and DCB simultaneously. After exclusion, 318 patients who matched the eligibility criteria were grouped into the DES (n = 57) and DCB (n = 261) groups and then compared retrospectively.

This retrospective study complied with the Declaration of Helsinki and was approved by the ethics committee of The First Affiliated Hospital of Zhengzhou University.

### Interventional procedures

Pre- and post-PCI, all patients were treated with dual antiplatelet therapy (DAPT) with oral aspirin (100 mg once daily) and clopidogrel (75 mg once daily) or ticagrelor (90 mg twice daily). In the case of emergent PCI, aspirin was administered at loading doses of 300 mg, and clopidogrel or ticagrelor was administered at 600 and 180 mg, respectively. The initial dosage of heparin (100 IU/kg) was administered before the procedure, and additional dosages were given to maintain an activated clotting time (ACT) > 300 s. RA was performed using the Rotablator™ (Boston Scientific, USA). The rotational speed ranged between 135,000 and 180,000 rpm, and the burr/vessel ratio was recommended at 0.4–0.6. Each RA time was 10–15 s. A continuous infusion “cocktail” containing verapamil, nitroglycerin, and heparin was administered to cool the RA system and avoid the slow flow phenomenon. After RA, predilatation using a semicompliant, noncompliant, or cutting balloon is left to the judgment of the operator in charge. If needed, several DESs or DCBs were applied to cover the whole stenotic segment. The DCB was dilated for at least 30–60 s at nominal pressure and used only in cases without flow-limiting dissection and severe vessel recoil. Upon procedure completion, intracoronary nitroglycerin was administered, and final angiography of the vessel was performed in at least two orthogonal views that showed the target site to be free of foreshortening or vessel overlap. Rescue DES implantation was recommended in the case of flow-limiting dissections after DCB treatment.

All patients in the RA/DCB group used paclitaxel-coated balloons (SeQuent Please, B. Braun, Melsungen, Germany). In the RA/DES group, the new-generation DES implanted included Excel (JW Medical System, China), Resolute Integrity™ (Medtronic, Santa Rosa, CA, USA), Excrossal (JW Medical System, China) and Synergy™ (Boston Scientific, Maple Grove, MN, USA).

### Follow-up and endpoints

Follow-up data were collected by telephone interviews after PCI and through the hospital's electronic medical record system when patients returned for further consultation. In-hospital events included all-cause mortality, MI, and stroke. In-hospital MI was defined as an increase in cTn values of more than 5 times within 48 h of PCI, along with either new ischemic ECG changes, pathological Q wave development, or angiographic evidence of a flow-limiting complication. The primary endpoint of the study was major adverse cardiovascular and cerebrovascular events (MACCE) at the follow-up, which was defined as the composite of all-cause death, nonfatal myocardial infarction, target lesion revascularization (TLR), and stroke.

### Statistical analysis

A paired-samples t test was used to compare data with regularly distributed quantitative data. With nonparametric data, the Wilcoxon matched-pair signed-rank test was used for analysis. The chi-squared test (Fisher's exact test) was used to examine qualitative data. The MACCE-free rates for the two groups were computed and graphically represented using the Kaplan‒Meier method. The multivariable Cox regression analysis model was built by stepwise selection. All analyses were conducted by SPSS26. *p* < 0.05 was considered statistically significant.

## Results

### Baseline clinical characteristics

A total of 318 patients who fulfilled the inclusion criteria were included in the study. Fifty-seven patients were treated with DCB after RA, and 261 were treated with DES. Baseline clinical characteristics for the two groups are shown in Table 1. The level of triglycerides (1.28 [0.92–1.78] mmol/L vs. 1.08 [0.83–1.39] mmol/L,* p* = 0.019) and LDL-c (1.94 [1.51–2.48] mmol/L vs. 1.78 [1.26–2.12] mmol/L,* p* = 0.019) were higher in the RA/DES group. The distribution of other clinical characteristics between the two groups was not different.

**Table 1:** Comparison of the two groups' baseline characteristics

Variable	RA/DCB (n = 57)	RA/DES (n = 261)	*t/χ*^*2*^*/Z*	*p*-value
Age (years)	66.68 ± 9.13	65.64 ± 7.99	0.803	0.424
Male, n (%)	30 (52.63)	154 (59.00)	0.779	0.377
Hypertension, n (%)	38 (66.67)	170 (65.13)	0.049	0.826
Diabetes mellitus, n (%)	24 (42.11)	96 (36.78)	0.564	0.453
Atrial fibrillation, n (%)	2 (3.51)	12 (4.59)	0.000	0.995
Stroke or TIA, n (%)	15 (26.32)	63 (24.14)	0.120	0.729
Chronic kidney disease, n (%)	4 (7.02)	29 (11.11)	0.460	0.498
Hyperlipidemia, n (%)	7 (12.28)	61 (23.37)	3.423	0.064
Prior MI, n (%)	11 (19.29)	42 (16.09)	0.346	0.556
Current smokers, n (%)	14 (24.56)	78 (29.89)	0.645	0.422
Prior PCI, n (%)	12 (21.05)	29 (11.11)	4.068	0.044
Prior CABG, n (%)	0 (0.00)	3 (1.15)	0.003	0.954
*Laboratory data*				
LVEF (%)	63.00 (60.00, 65.00)	62.00 (60.00, 64.00)	1.084	0.278
Hemoglobin, g/L	124.56 ± 17.32	125.43 ± 17.45	0.342	0.733
HbA1c (%)	6.10 (5.7, 7.19)	6.10 (5.60, 7.07)	0.174	0.862
eGFR, ml/min/1.73m^2^	87.00 (76.50, 96.00)	89.00 (78.00, 96.00)	0.005	0.966
Total cholesterol, mmol/L	3.29 (2.69, 3.75)	3.38 (2.95, 4.04)	1.941	0.052
Triglycerides, mmol/L	1.08 (0.83, 1.39)	1.28 (0.92, 1.78)	2.348	0.019
HDL-c, mmol/L	1.29 (1.04, 1.54)	1.19 (1.01, 1.39)	1.277	0.202
LDL-c, mmol/L	1.78 (1.26, 2.12)	1.94 (1.51, 2.48)	2.244	0.025
Glucose, mg/dL	127.96 ± 35.38	126.05 ± 36.47	0.359	0.720
SYNTAX score	25.00 (17.00, 30.00)	25.00 (19.00, 33.00)	1.236	0.217

MI, myocardial infarction; PCI, percutaneous coronary intervention; CABG, coronary artery bypass graft; LVEF, left ventricular ejection fraction; HbA1c, Glycosylated hemoglobin; eGFR, estimated glomerular filtration rate; HDL-c, high density lipoprotein cholesterol; LDL-c, low density lipoprotein cholesterol

### Lesions and procedural characteristics

There were 332 consecutive lesions from 318 patients who underwent RA. Table 2 provides a summary of the lesions and procedural characteristics of the two groups. The number of left main and bifurcation lesions was larger in the RA/DES group, as were the number of implants, implant diameter, and total implant length. The utilization of IVUS/OCT, maximum burr size, and burr size and reference diameter were all significantly higher in the RA/DCB group.

**Table 2:** Comparison of the two groups' lesion characteristics and surgical data (332 lesions)

Variable	RA/DCB (n = 58)	RA/DES (n = 274)	*χ*^*2*^*/Z*	*p*-value
*Approach*			0.547	0.460
Via radial artery, n (%)	49.00 (84.48)	220 (80.29)		
Via femoral artery, n (%)	9.00 (15.52)	54 (19.71)		
*Sheath size, French (F)*			0.194	0.659
6F, n (%)	28.00 (48.28)	141 (51.46)		
7F, n (%)	30.00 (51.72)	133 (48.54)		
*Target-vessel, n (%)*			24.610	< 0.001
LM, n (%)	1 (1.72)	67 (24.45)		
LAD, n (%)	42 (72.41)	152 (55.47)		
LCX, n (%)	7 (12.07)	13 (4.74)		
RCA, n (%)	8 (13.79)	42 (15.33)		
CTO, n (%)	6 (10.34)	25 (9.12)	0.084	0.772
Bifurcation, n (%)	2 (3.45)	51 (18.61)	7.114	0.008
Triple vessel disease, n (%)	20 (34.48)	119 (43.43)	1.575	0.210
IVUS/OCT, n (%)	34 (58.62)	106 (38.69)	7.800	0.005
Temporary peacemaker, n (%)	2 (3.45)	18 (6.57)	0.365	0.546
IABP, n (%)	1 (1.72)	10 (3.65)	0.116	0.733
Number of burrs, n	1.17 ± 0.43	1.29 ± 0.53	1.589	0.112
Largest burr size, mm	1.60 ± 0.19	1.54 ± 0.16	2.502	0.012
Burr/artery ratio	0.61 ± 0.09	0.52 ± 0.06	7.063	< 0.001
Number of devices, n	1.57 ± 0.65	2.17 ± 0.79	5.406	< 0.001
Diameter of device, mm	2.68 ± 0.38	2.99 ± 0.34	5.695	< 0.001
Total length of device, mm	42.60 ± 17.42	52.16 ± 20.76	3.521	< 0.001

LM, left main artery; LAD, left anterior descending artery; LCX, left circumflex artery; RCA, right coronary artery; CTO, chronic total occlusion; IVUS, intravascular ultrasound; OCT, optical coherence tomography; IABP, intra-aortic balloon pump

### Procedural complications, in-hospital events, and clinical follow-up

The follow-up ended in May 2022. The follow-up period of the RA/DCB group was 15.0 (12.0, 22.5) months, while that of the RA/DES group was 22.0 (15.0, 30.0) months. Two patients in the RA/DCB group underwent rescue DES implantation due to severe dissection after DCB implantation. Major procedural complications (8.77% vs. 8.43% *p* = 0.933) and in-hospital events (0.00% vs. 2.68% *p* = 0.452) did not differ between the two groups. Duration of DAPT (12.28 ± 2.41 months vs. 10.25 ± 3.53 months, *p* = 0.019) were higher in the RA/DES group. MACCE (18.77% vs. 12.28%) and TLR (13.79% vs. 7.02%) occurred more frequently in the RA/DES group, but the differences were not statistically significant (*p* > 0.05). Details are listed in Table 3.

**Table 3:** Comparison of the two groups' intraoperative complications, in-hospital adverse events and long-term follow-up results

Variable	RA/DCB (n = 57)	RA/DES (n = 261)	*t*/*χ*^2^/*Z*	*p*-value
Procedural complications, n (%)	5 (8.77)	22 (8.43)	0.007	0.933
Slow or no-reflow, n (%)	2 (3.51)	16 (6.13)	0.211	0.646
Perforation, n (%)	0 (0.00)	2 (0.77)	0.000	1.000
Burr entrapment, n (%)	1 (1.75)	1 (0.38)	0.068	0.794
Guidewire break, n (%)	1 (1.75)	2 (0.77)	0.000	1.000
Hypotension, n (%)	1 (1.75)	1 (0.38)	0.068	0.794
In-hospital events, n (%)	0 (0.00)	7 (2.68)	0.566	0.452
All-cause mortality, n (%)	0 (0.00)	4 (1.53)	0.081	0.776
Myocardial infarction, n (%)	0 (0.00)	2 (0.77)	0.000	1.000
Stroke, n (%)	0 (0.00)	1 (0.38)	0.000	1.000
Long-term MACCE, n (%)	7 (12.28)	49 (18.77)	1.359	0.244
All-cause mortality, n (%)	1 (1.75)	4 (1.53)	0.000	1.000
Myocardial infarction, n (%)	0 (0.00)	3 (1.15)	0.003	0.954
TLR, n (%)	4 (7.02)	36 (13.79)	1.386	0.239
Stroke, n (%)	2 (3.51)	6 (2.29)	0.004	0.951
Duration of DAPT, m	10.25 ± 3.53	12.28 ± 2.41	5.241	< 0.001
Follow-up CAG rate, n (%)	29 (50.88)	122 (46.74)	0.321	0.571
Follow-up duration, m	15.0 (12.0, 22.5)	22.0 (15.0, 30.0)	4.014	< 0.001

MACCE, major adverse cardiovascular and cerebrovascular events; TLR, target lesion revascularization; DAPT, duration of dual antiplatelet therapy; CAG, coronary angiography

### Association between RA and MACCE

**Fig. 1:**
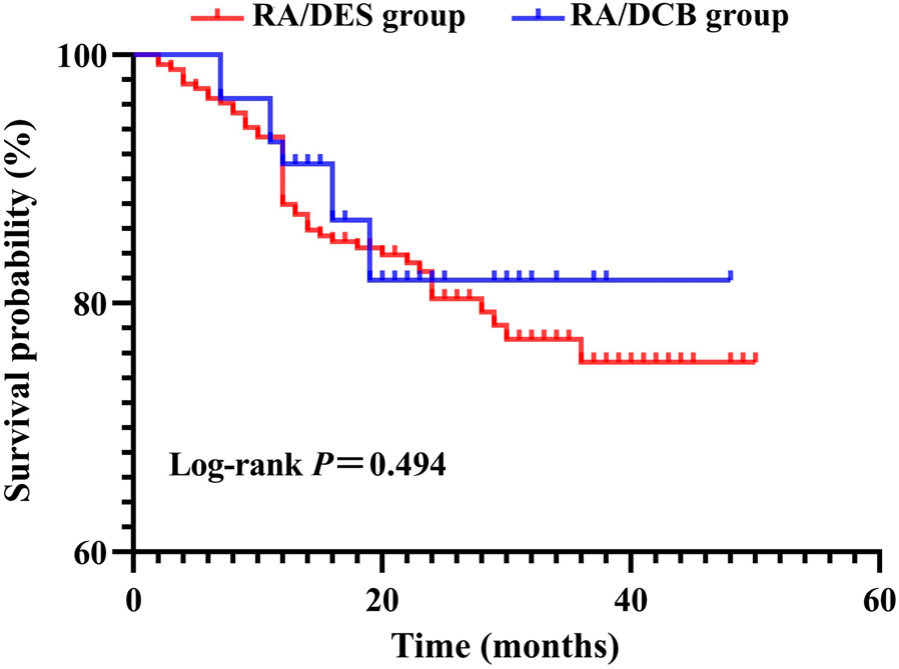
Kaplan–Meier curve of MACCE free survival

Both groups had the same Kaplan–Meier curve for the cumulative incidence of MACCE (Fig. 1). Table 4 shows details of the multivariate Cox regression analysis that adjusts for significant variables in univariate testing. Bifurcation lesions (HR 2.284, 95% CI 1.063–4.908, *p* = 0.034), total length of DCB/DES (HR 1.023, 95% CI 1.005–1.047, *p* = 0.014) and SYNTAX score (HR 1.047, 95% CI 1.013–1.082, *p* = 0.006) were independent risk factors for MACCE during the follow-up.

**Table 4:** Univariate and multivariate Cox regression analysis

Variable	Univariate analysis		Multivariate analysis	
	HR (95% CI)	*p-*value	HR (95% CI)	*p-*value
Prior CABG	0.188 (0.046–0.775)	0.021	1.136 (0.206–6.265)	0.884
Total cholesterol	1.398 (1.043–1.873)	0.025	1.300 (0.961–1.759)	0.089
LM lesions	0.504 (0.290–0.877)	0.015	0.740 (0.338–1.620)	0.451
Triple vessel disease	0.482 (0.282–0.825)	0.008	1.118 (0.597–2.095)	0.728
Bifurcation lesions	0.392 (0.222–0.694)	0.001	2.284 (1.063–4.908)	0.034
Number of devices	1.431 (1.037–1.974)	0.029	0.807 (0.491–1.327)	0.398
Total length of device	1.021 (1.009–1.034)	0.001	1.023 (1.005–1.047)	0.014
SNYTAX score	1.065 (1.038–1.093)	0.001	1.047 (1.013–1.082)	0.006

## Discussion

The present study suggests that the efficacy and safety of the RA/DCB strategy for CAC might be comparable to those of DES implantation, although significant differences in lesion features between the two groups at baseline. Cox regression analysis showed that DCB implantation was not associated with increased long-term MACCE of RA-PCI. Bifurcation lesions, SYNTAX scores, and the total length of device were independent predictors of MACCE. Based on the above results, it can be concluded that the severity of the lesions affects the long-term prognosis of patients who underwent RA-PCI.

Although there is a significant correlation between CAC and coronary events, other prevalent cardiovascular risk factors do not appear indispensable for its progression. The genetic component thus appears to be important. By promoting the osteogenic differentiation of vascular smooth muscle cells, for instance, the β2-AR and G protein pathways may be crucial in forming calcified plaques [6]. Currently, severe CAC remains a challenge for successful PCI, which often requires the assistance of RA to modify the calcified plaque. Currently, RA followed by DES is the standard PCI strategy for CAC patients. However, there are certain limitations in the application of DES in CAC. First, heavily calcified plaques impair the trabecular meshwork, disrupting drug-polymer from the stent surface and reducing the success rate and long-term effectiveness of PCI [7]. Second, the existence of calcified nodules impedes complete stent expansion and accurate apposition, leading to an increased rate of in-stent restenosis and stent thrombosis [8]. Despite being rare, stent thrombosis may cause myocardial infarction (MI) or cardiac death [9]. Additionally, diffuse lesions are common in calcified vessels, which frequently need longer and numerous DES implanted to cover completely [10]. In this study, there were also many diffuse lesions, and the total length of devices in RA/DES group reached 52.16 ± 20.76 mm. Research shows that the implantation of lengthy stents may hinder the stented segment's ability to regain vasomotion, encourage neoatherosclerosis, and restrict access to revascularization [11].

The strategy of RA followed by DCB has numerous potential benefits in CAC. (1) RA can efficiently cleave calcified circles, lowering the danger of severe dissections brought by high-pressure dilation [12]. Moreover, elastic recoil does not easily occur in post-RA calcified vessels (mechanical support of the calcified circle). In previous studies, 6–27% of noncalcified vessels were implanted with rescue DES due to severe dissection or elastic recoil after DCB implantation [13]. (2) DCB-based PCI without leaving metal residues avoids the risk of stent thrombosis and leads to shorter dual antiplatelet therapy [14]. (3) In calcified vessels with diffuse long lesions, the use of DCB can avoid the implantation of lengthy stents and maintain access for future revascularization when needed. (4) For individuals with complex anatomical features such as ostial lesions, bifurcation lesions, and diffuse lesions, DCB implantation is typically more accessible and faster than DES implantation.

Previous studies on RA followed by DES showed that the target vessel revascularization (TLR) rate varied between 6.8 and 11.7% [15–17]. In this study, the TLR rate in the RA/DES group was 13.79%, which was slightly higher than that in previous reports, and the incidence of TLR in the RA/DCB group was 7.02%, similar to the previous results of RA combined with DES. The results of RA combined with DCB were reported by Rissanen et al. [18] showed that the TLR rate was extremely low (1.5% at 12 months), which may be related to the low follow-up rate of angiography. Three small sample studies from Japan showed that the TLR rate is 6–16%, which is close to the results of our study [12, 19, 20]. Currently, there are few studies on the application of DCBs in CAC. A retrospective study revealed no significant difference in MACCE, TLR rate, and late lumen loss between the calcified and noncalcified groups after DCB implantation [13]. In terms of safety, it is worth mentioning that in this study, DCBs were implanted in only a few patients, while DESs were implanted in more patients with severe dissection or high residual stenosis. In the RA/DCB group, two patients underwent rescue DES implantation after DCB implantation due to severe dissection, and no acute occlusion occurred. Compared with the RA/DES group, there was no significant difference in complications and in-hospital adverse events. Therefore, we believe that DCB implantation is safe when RA effectively relieves the burden of calcified plaque without causing severe dissection.

Coronary angiography only offers two-dimensional pictures, which places various limitations in evaluating calcified lesions. Intracoronary imaging modalities such as OCT and IVUS are three-dimensional imaging tools that can precisely evaluate calcified lesions and display the intima, media, and adventitia of coronary vessels [21]. Additionally, it can offer comprehensive instructions for choosing the right rotary burr size and developing an RA scheme. Studies show that intracoronary imaging guidance can improve the success rate of PCI, obtain more significant luminal gain and improve long-term prognosis [22]. The predilation requirements for DCBs are higher than those for DESs. Intracoronary imaging mode can accurately evaluate the predilate results, select the appropriate DCB size and accurately position the DCB to achieve complete adhesion and coverage of lesions. In addition, dissection after DCB implantation is usually inevitable. Mild to moderate dissection that does not affect blood perfusion is usually safe, and it can encourage the drug to penetrate the vessel wall, thus promoting long-term lumen enlargement [23]. However, for severe dissection, due to the lack of stent support, the dissection may expand distally and cause vascular occlusion. Coronary angiography is less sensitive to dissection, often missed or underestimated [24]. Intravascular imaging can sensitively detect severe dissection that can lead to acute vascular occlusion and guide the implantation of rescue stents [25]. In this study, approximately 58.62% of patients in the RA/DCB group received PCI under the guidance of intracoronary imaging, which was higher than the 38.69% in the RA/DES group. It is worth mentioning that in the RA/DCB group, after DCB implantation, IVUS detected a high-risk dissection that was not found by angiography, and then a rescue DES was implanted.

There are some limitations to this research. First, this was a single-center retrospective study in which the operator decided on the implantation of DCB or DES after RA. Second, this study only compared the curative effects of DCB and DES alone after RA but did not include patients who used DCB and DES simultaneously. Third, baseline lesion characteristics was quite different between DCB and DES groups, DCB tended to be used for simpler cases in severe calcified lesions. Fourth, the number of patients in the DCB group was small, and the number of patients who received coronary angiography follow-up after PCI was limited. In the future, more large-sample randomized controlled studies are expected to clarify the safety and effectiveness of RA combined with DCB in treating severe coronary artery calcification.

## Conclusion

The results of this study suggest that stent-less RA/DCB PCI offers a viable alternative for severe coronary artery calcification and may be a promising interventional therapy.

## Data Availability

The datasets used and/or analysed during the current study available from the corresponding author on reasonable request.

## Abbreviations

DCB: Drug-coated balloons
RA: Rotational atherectomy
CAC: Coronary artery calcification
PCI: Percutaneous coronary intervention
DES: Drug-eluting stents
DAPT: Dual antiplatelet therapy
MACCE: Major adverse cardiovascular and cerebrovascular events
TLR: Target vessel revascularization
IVUS: Intravascular ultrasound
OCT: Optical coherence tomography

## References

[1] Huang BT, Huang FY, Zuo ZL, Liu W, Huang KS, Liao YB, Wang PJ, Peng Y, Zhang C, Zhao ZG (2015). Target lesion calcification and risk of adverse outcomes in patients with drug-eluting stents. A meta-analysis. Herz.

[2] Demuyakor A, Hu S, Koniaeva E, Liu M, Weng Z, Zhao C, Feng X, He L, Xu Y, Zeng M (2022). Impact of nodular calcification in patients with acute coronary syndrome (ACS) treated with primary percutaneous coronary intervention (PCI). BMC Cardiovasc Disord.

[3] Sharma SK, Tomey MI, Teirstein PS, Kini AS, Reitman AB, Lee AC, Généreux P, Chambers JW, Grines CL, Himmelstein SI (2019). North American expert review of rotational atherectomy. Circ Cardiovasc Interv.

[4] Buccheri D, Lombardo RM, Cortese B (2019). Drug-coated balloons for coronary artery disease: current concepts and controversies. Future Cardiol.

[5] Jeger RV, Eccleshall S, Wan Ahmad WA, Ge J, Poerner TC, Shin ES, Alfonso F, Latib A, Ong PJ, Rissanen TT (2020). Drug-coated balloons for coronary artery disease: third report of the international DCB consensus group. JACC Cardiovasc Interv.

[6] Gambardella J, Wang X, Mone P, Khondkar W, Santulli G (2020). Genetics of adrenergic signaling drives coronary artery calcification. Atherosclerosis.

[7] Kaul A, Dhalla PS, Bapatla A, Khalid R, Garcia J, Armenta-Quiroga AS, Khan S (2020). Current treatment modalities for calcified coronary artery disease: a review article comparing novel intravascular lithotripsy and traditional rotational atherectomy. Cureus.

[8] Abusnina W, Mostafa MR, Al-Abdouh A, Radaideh Q, Ismayl M, Alam M, Shah J, Yousfi NE, Paul TK, Ben-Dor I (2022). Outcomes of atherectomy in treating severely calcified coronary lesions in patients with reduced left ventricular ejection fraction: a systematic review and meta-analysis. Front Cardiovasc Med.

[9] Park KH, Jeong MH, Hong YJ, Ahn Y, Kim HK, Koh YY, Kim DI, Kim SW, Kim W, Rha SW (2018). Effectiveness and safety of biolimus A9™-eluting stEnt in patients with AcUTe coronary sYndrome; a multicenter, observational study (BEAUTY study). Yonsei Med J.

[10] Ma X, Chen P, Zhao Y, Zeng C, Xin M, Ye Q, Wang J (2019). Coronary angiography characteristics of symptomatic patients with prior coronary artery bypass graft: a descriptive study. Biomed Res Int.

[11] Díaz Fernández JF, Camacho Freire SJ, Fernández Guerrero JC, Delarche N, Bretelle C, Zueco Gil J, Palop RL, García Del Blanco B, Mainar Tello V, Albert F (2018). Everolimus drug-eluting stent performance in patients with long coronary lesions: the multicenter Longprime registry. Catheteriz Cardiovasc Intervent.

[12] Ueno K, Morita N, Kojima Y, Takahashi H, Kawasaki M, Ito R, Kondo H, Minatoguchi S, Yoshida T, Hashimoto Y. Safety and long-term efficacy of drug-coated balloon angioplasty following rotational atherectomy for severely calcified coronary lesions compared with new generation drug-eluting stents. J Intervent Cardiolo. 2019. 10.1155/2019/9094178 PMC6739772 31772551

[13] Ito R, Ueno K, Yoshida T, Takahashi H, Tatsumi T, Hashimoto Y, Kojima Y, Kitamura T, Morita N (2018). Outcomes after drug-coated balloon treatment for patients with calcified coronary lesions. J Interv Cardiol.

[14] Ho HH, Lee JH, Khoo DZL, Hpone KKS, Li KFC (2021). Shockwave intravascular lithotripsy and drug-coated balloon angioplasty in calcified coronary arteries: preliminary experience in two cases. J Geriatric Cardiol.

[15] Abdel-Wahab M, Baev R, Dieker P, Kassner G, Richardt G (2013). Long-term clinical outcome of rotational atherectomy followed by drug-eluting stent implantation in complex calcified coronary lesions. Catheteriz Cardiovasc Intervent.

[16] Abdel-Wahab M, Richardt G, Joachim Büttner H, Toelg R, Geist V, Meinertz T, Schofer J, King L, Neumann FJ, Khattab AA (2013). High-speed rotational atherectomy before paclitaxel-eluting stent implantation in complex calcified coronary lesions: the randomized ROTAXUS (rotational atherectomy prior to taxus stent treatment for complex native coronary artery disease) trial. JACC Cardiovasc Interv.

[17] Tian W, Mahmoudi M, Lhermusier T, Pendyala LK, Kiramijyan S, Saar M, Waksman R (2015). Clinical outcomes of first- and second-generation drug-eluting stents in patients undergoing rotational atherectomy for heavily calcified coronary lesions. Cardiovasc Revasc Med Incl Mol Interv.

[18] Rissanen TT, Uskela S, Siljander A, Kärkkäinen JM, Mäntylä P, Mustonen J, Eränen J (2017). Percutaneous coronary intervention of complex calcified lesions with drug-coated balloon after rotational atherectomy. J Interv Cardiol.

[19] Shiraishi J, Kataoka E, Ozawa T, Shiraga A, Sawada T. Angiographic and clinical outcomes after stent-less coronary intervention using rotational atherectomy and drug-coated balloon in patients with de novo lesions. Cardiovasc Revasc Med. 2019. 10.1016/j.carrev.2019.08.020 31494063

[20] Nagai T, Mizobuchi M, Funatsu A, Kobayashi T, Nakamura S (2020). Acute and mid-term outcomes of drug-coated balloon following rotational atherectomy. Cardiovasc Interv Ther.

[21] Teng W, Li Q, Ma Y, Cao C, Liu J, Zhao H, Lu M, Hou C, Wang W (2021). Comparison of optical coherence tomography-guided and intravascular ultrasound-guided rotational atherectomy for calcified coronary lesions. BMC Cardiovasc Disord.

[22] Darmoch F, Alraies MC, Al-Khadra Y, Moussa Pacha H, Pinto DS, Osborn EA (2020). Intravascular ultrasound imaging-guided versus coronary angiography-guided percutaneous coronary intervention: a systematic review and meta-analysis. J Am Heart Assoc.

[23] Cortese B, Silva Orrego P, Agostoni P, Buccheri D, Piraino D, Andolina G, Seregni RG (2015). Effect of drug-coated balloons in native coronary artery disease left with a dissection. JACC Cardiovasc Interv.

[24] Ali ZA, Maehara A, Généreux P, Shlofmitz RA, Fabbiocchi F, Nazif TM, Guagliumi G, Meraj PM, Alfonso F, Samady H (2016). Optical coherence tomography compared with intravascular ultrasound and with angiography to guide coronary stent implantation (ILUMIEN III: OPTIMIZE PCI): a randomised controlled trial. Lancet (London, England).

[25] Peng X, Qu W, Jia Y, Wang Y, Yu B, Tian J (2020). Bioresorbable scaffolds: contemporary status and future directions. Front Cardiovasc Med.

